# The role of dental pain and psychosocial factors on the relationship between dental caries and oral health-related quality of life in children

**DOI:** 10.1186/s12903-022-02372-2

**Published:** 2022-08-10

**Authors:** Cilio Antonio Ribeiro Junior, Mario Vianna Vettore, Janete Maria Rebelo Vieira, Ana Paula Corrêa de Queiroz Herkrath, Adriana Corrêa de Queiroz Herkrath, Juliana Vianna Pereira, Fernando José Herkrath, Maria Augusta Bessa Rebelo

**Affiliations:** 1 School of Dentistry, Federal University of Amazonas, Av. Ministro Waldemar Pedrosa, 1539, Praça 14 de Janeiro, Manaus, AM CEP 69025-050 Brazil; 2 Department of Health and Nursing Sciences, Faculty of Health and Sports Sciences, University of Agder, Campus Kristiansand, Universitetsveien 25, 4630 Kristiansand, Norway; 3 Instituto Leônidas e Maria Deane, Fundacão Oswaldo Cruz, Rua Teresina, 476, Adrianópolis, Manaus, AM CEP: 69027-070 Brazil

**Keywords:** Dental caries, Dental pain, Sense of coherence, Social support, Quality of life

## Abstract

**Background:**

To examine the role of dental pain, sense of coherence (SOC) and social support on the relationship between dental caries and oral health-related quality of life (OHRQoL) in children aged 12 years.

**Methods:**

A cross-sectional study involving 400 schoolchildren selected from public schools in a socioeconomically disadvantaged region in the city of Manaus, Brazil was carried out. The predictors of OHRQoL were selected according to the Wilson and Cleary theoretical model, including number of decayed teeth and its clinical consequence (component D of the DMFT index and PUFA/pufa index), dental pain (symptom status), and SOC and social support (individual and environmental characteristics). Statistical analysis was conducted through structural equation modelling and multivariable negative binomial regression. The significance level established for all analyses was 5%.

**Results:**

Number of dental caries was indirectly linked with OHRQoL (β = 0.19, 95% CI 0.11/0.29) through dental pain, SOC and social support. Clinical consequences of untreated caries directly predicted poor OHRQoL (β = 0.12, 95% CI 0.01/0.23). Dental pain, SOC and social support did not moderate the effect of dental caries measures on OHRQoL.

**Conclusion:**

Our findings suggest the role of dental pain, SOC and social support as mediator factors on the link between dental caries and OHRQoL. Tackling dental caries along with psychosocial factors may attenuated the impact of oral health on OHRQoL in children.

## Background

Oral health-related quality of life (OHRQoL) is considered a relevant and contemporary health outcome in dental research that assess the individual perceptions of the impact of dental problems on functional, psychological and social aspects of quality of life [1, 2]. It has been acknowledged that enhancing the understanding of the predictors of OHRQoL is relevant to promote oral health in order to alleviate the impact of oral diseases on people’s daily life and well-being.

Evidence suggests the role of clinical factors, symptom and functional status, and individual and environmental factors on OHRQoL [3, 4]. More specifically, poor dental clinical measures have been associated with worse OHRQoL in adolescents [3–6]. Of them, dental caries has been consistently reported as a predictor of OHRQoL through direct or indirect effects [3–6]. These findings highlight the importance of dental caries as oral condition of a dental public health importance.

The Global Burden of Disease study revealed that 30% of the global population has dental caries in permanent teeth [7]. In addition, despite the oral health improvements in most countries, dental caries disparities across socioeconomic groups are persistent problems in children [7]. The impact of dental caries on daily performance is noteworthy among children and adolescents. For instance, dental caries was associated with daily activities in adolescents, and such association differed between socioeconomic groups [8]. Furthermore, a recent systematic review showed that dental caries in children and adolescents was a meaningful determinant of poor school performance and poor school attendance [9].

The relationship between dental caries and OHRQoL is complex because of the importance of individual and environmental factors as well as the possible role of mediators between predictors and OHRQoL. The Wilson and Cleary model provides a sound theoretical framework to examine the predictors of OHRQoL outcomes as well to explore the direct and indirect links between them [10]. The model displays the relationships between measures schematically defining a priori possible intervening variables between biological and physiological factors, and quality of life, including symptom status and functional status. The model also acknowledges that other relationships between nonadjacent variables might be considered [10]. Previous studies using the Wilson and Cleary model demonstrated that dental caries was directly associated with poor OHRQoL [6, 8]. In addition, the indirect effect of dental caries on OHRQoL was observed [3–5, 8]. The pathways by which dental caries influenced OHRQoL included symptoms (e.g. dental pain) [3, 4], functional status [3], individual characteristics (e.g. psychological characteristics) [5], and environmental factors (eg. socioeconomic position) [8].

Previous studies have given little attention on the potential mediators and moderators on the link between dental caries and OHRQoL. The dearth of studies investigating the possible mediators and moderators in such relationship suggests a relevant gap in the dental literature to understand the interrelationships between dental clinical measures and OHRQoL. The possible role of dental symptoms (e.g. dental pain), and psychosocial factors, including sense of coherence (SOC) and social support as mediators on the link between dental caries and OHRQoL has been little explored in dental research [4, 5]. Additional research examining the potential pathways between dental caries and OHRQoL as well as the effect modification of the predictors of OHRQoL may provide new insights on the potential mediators and effect modifiers that may influence the extent and nature of the abovementioned relationship, which are needed to orientate clinical practices and oral health policies.

It has been claimed that studies not examining mediators and effect modifiers on the predictors of OHRQoL may lose the explanatory power to represent the reality, since a given measured effect is eliminated from its context [11]. Therefore, the aim of this study was to evaluate the mediation and effect modification of dental pain, SOC and social support on the relationship between dental caries and OHRQoL in 12-yeard-old schoolchildren.

## Methods

### Study design and participants

This was a cross-sectional study involving 12-year-old children from a region with low socioeconomic indicators in the city of Manaus (East Zone), Brazil. The city of Manaus is the capital of Amazonas state, located in the North region of Brazil. The Human Development Index of Manaus and the East Zone of the city was 0.737 and 0.659 in 2010. A randomized stratified sample of public schools with children in the 7th year from primary school classes was selected in the 11 neighbourhoods that make up the eastern region of the city of Manaus. In the two-stage probabilistic sampling, the schools were proportionally selected according to the corresponding number of schools in each neighbourhood.

All 12-year-olds in the 7th year from all classes of the selected schools were invited to participate in the study. Children in the 7th year of selected schools who were not 12 years-old and those aged 12 years but not attending the 7th year were not invited to participate. Exclusion criteria were use of orthodontic appliances, children with syndromes and those with special care needs.

### Study power calculation

The present study included a final sample size of 400 schoolchildren, which resulted in a study a power of 90% with 5% of statistical significance to detect effects of 0.10 using structural equation modelling analysis with four observed variables and two latent variables [12]. In addition, a sample of 400 schoolchildren would lend a power of 95% in a multiple regression model including 3 predictors considering a statistical significance of 5% to detect effects of 0.05.

### Theoretical model

**Fig. 1:**
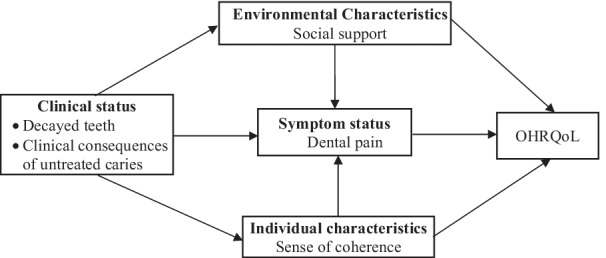
Theoretical model adapted from Wilson and Cleary [10]

The theoretical model used in this study was adapted from the conceptual model proposed by Wilson and Cleary model [10]. The model included biological and physiological factors (number of decayed teeth and number of teeth presenting clinical consequences of untreated dental caries), symptom status (dental pain), individual characteristics (sense of coherence), environmental characteristics (social support) and quality of life related to oral health (OHRQoL). Dental pain, SOC and social support were considered the possible mediators and effect modifiers on the relationship between dental caries and OHRQoL (Fig. 1).

### Data collection and measures

Data were collected through dental clinical examinations and pre-tested questionnaires carried out by five previously calibrated dentists from October 2016 to June 2017.

A questionnaire on socioeconomic characteristics, including parent’s/guardian’s level of education (years of study) and monthly family income (Up to ½ Brazilian Minimal Wage [BMW], ½ to 1 BMW, > 1 BMW) was completed by children’s parents/guardians. One BMW corresponded to U$271.09 in 2016. Children answered a self-completed questionnaire to assess sex, dental pain, SOC, social support and OHRQoL.

The schoolchildren were examined under natural light on the premises of the selected schools. First, supervised dental brushing using toothbrush, fluoride dentifrice and dental floss was performed. Then, clinical examination was conducted using a plain dental mirror No. 5 (Duflex®) and a ball point OMS (Stainless®) probe. Schoolchildren were examined sitting in school chairs.

### Oral health-related quality of life

Child Perceptions Questionnaire (CPQ_11-14_) Impact Short Form (ISF: 16) was used to evaluate children’s OHRQoL [13]. The questionnaire consists of 16 items grouped into 4 dimensions: oral symptoms, functional limitations, emotional state and social well-being, that evaluate the frequency of life events during the last 3 months. A 5-point Likert scale was used for each response: 0 = Never; 1 = Once or twice; 2 = Sometimes; 3 = Often; 4 = Every day or almost every day. The CPQ_11-14_ total score is obtained by summing the items and can range from 0 to 64. The higher the score the greater the impact of oral health status on children’s quality of life.

### Dental caries

Dental caries was measured according to the number of decayed teeth [14] and clinical consequences of untreated dental caries (PUFA/pufa index) [15]. The former is obtained by adding the total number of permanent teeth presenting clinical cavities due to caries, based on the component decayed of the decayed, missing and filled teeth (DMFT) index [14]. PUFA/pufa index was used to assess the occurrence of dental conditions resulting from untreated dental caries including visible pulp, ulceration of the oral mucosa due to root fragments, fistula, or abscess. The number of teeth with clinical consequences of untreated dental caries are summed to obtain the PUFA/pufa index [15].

### Mediators

The mediators included dental pain, SOC and social support. Dental pain was assessed according to the following question: “Did you experience toothache during the last 6 months?”, using the response options 0 = No or 1 = Yes. The 6-months period prevalence of dental pain was used because OHRQoL measure (CPQ_11-14_) referred to the last 3 months. Thus, dental pain should be reported for period longer than 3 months according to the theoretical model (Fig. 1.) A period prevalence of dental pain longer than 6 months would possibly result in recall bias due to the age of the participants.

Children’s SOC was measured using the SOC-13 scale [16] transculturally adapted to the Portuguese language [17]. SOC-13 scale consists of 13-item questionnaire using a 5-point Likert scale. The scores of the items related to negative SOC were reversed before adding the scores to obtain the final SOC score. The final score can range from 13 to 65 points. The higher the final score the greater the SOC.

Social support was evaluated through of the Social Support Appraisals (SSA) scale validated for Brazilian participants [18]. The SSA scale is a 30-item questionnaire answered using a 6-point Likert scale using the following response options: 1 = I fully agree, 2 = I strongly agree, 3 = I agree a little, 4 = I disagree somewhat, 5 = I strongly disagree, 6 = I fully disagree. The items are grouped into four dimensions of social support: family, friends, teachers and others. The total SSA score results from the sum of the 30 items, ranging from 30 to 180. The items against social support are reversed before obtaining the SSA score. Thus, the higher the SSA score indicates greater social support.

### Calibration study and reliability analysis during the main study

Initially, five dentists were calibrated for dental examinations previous to data collection of the main study. The calibration study included 20 children from the same schools who did not participate in the main study. They were examined twice on 7 days interval according to the same dental exam protocol and instruments used in the main study to obtain repeated measures of DMFT index and PUFA/pufa index. The intra-examiner Kappa coefficient for DMFT index ranged from 0.80 to 0.81 and for PUFA/pufa index ranged from 0.60 to 0.90. The Kappa coefficient for inter-examiner agreement ranged from 0.90 to 1.00 for DMFT index, and from 0.60 to 0.90 for PUFA/pufa index.

Cronbach’s alpha of CPQ_11-14_, SOC-13 and SSA scales was 0.674, 0.876 and 0.812, respectively. Dental examinations and questionnaires were replicated in 10% of the sample in the main study over a 14-day period. The intra-examiner Kappa coefficients for DMFT index and PUFA/pufa index were 0.93 and 0.87, respectively. Intra-Class Correlation Coefficient for CPQ_11-14_, SOC-13 and SSA scales were 0.83, 0.89 and 0.89, respectively.

### Data analysis

Data analysis was carried out in three steps. First, the variables were described using frequencies and means (standard deviations) for categorical and continuous variables.

Second, confirmatory factorial analysis (CFA) and structural equation modelling (SEM) were used to evaluate mediation. CFA evaluated the measurement model concerning multidimensionality of the two latent variables and the respective indicators [19]. OHRQoL was a latent variable composed by 4 indicators represented by the CPQ_11-14_ dimensions: oral symptoms, functional limitations, emotional state and social well-being. Social support was a latent variable created from four indicators represented by the SSA dimensions: family, friends, teachers and others. SEM assessed mediation through the direct and indirect relationships between observed and latent variables according to the theoretical model (Fig. 1). The total effects representing the sum of the direct and indirect effects, were obtained using the maximum likelihood estimation method. Mediation was detected when the indirect effect between two variables (e.g. dental caries and OHRQoL) was significant. In this case, the variables representing the different pathways (e.g. SOC, social support, dental pain) between the exposure (e.g. dental caries) and the outcome (e.g. OHRQoL) were considered the mediators even when the direct relationship between the exposure and the mediator, or between the mediator and the outcome was not significant. Sex and monthly family income were included in the SEM for adjustment. Nine hundred samples via bootstrap procedure were re-sampled from the original dataset to estimate the 95% confidence intervals (CI) and more accurate standard errors [20]. The Chi-square test (χ^2^/df < 3.0) was used to assess the adequacy of the overall fit of the model. In addition, the following fit indexes and thresholds were used to assess the model fit. GFI (Goodness of Fit) ≥ 0.90, CFI (Comparative Fit Index) ≥ 0.90, SRMR (Standardized Root Mean Square Residual) ≤ 0.08 and RMSEA (Root Mean Square Error of Approximation) ≤ 0.06 [21]. The CFA and SEM analyzes were performed using AMOS 25.0.

Third, moderating effect analysis of the interaction of dental pain, SOC and social support with dental caries on OHRQoL were tested using negative binominal regression according to each moderation variable. Initially, the likelihood ratio test was used to compare the Akaike’s information criterion (AIC) of the null negative binomial regression model with the school-level variable (AIC = 2801.946) and without the school-level variable (AIC = 2802.842). Multilevel analysis accounting for school-level was not used since both models were not statistically different (*P* = 0.639). Three statistical models were tested for each moderation variable. The model 1 tested the crude association between of dental caries (number of decayed teeth and number of teeth with clinical consequences of untreated dental caries) and OHRQoL. In model 2, the moderation variable (dental pain, SOC or social support), sex and monthly family income were inserted in the regression model. Model 3 included the variables in model 2 and the interaction term “dental caries × moderation variable”. The assessment of the interaction effect was by comparing the Likelihood Ratio between model 2 (without the interaction term) and model 3 (with the interaction term) using the Chi-square test. Statistical differences between model 2 and Model 3 suggested moderating effect. The analyzes of moderation effect were performed using STATA 14.0.

### Ethic aspects

This research was conducted in accordance with the Helsinki Declaration and approved by the Ethics Committee of the Federal University of Amazonas (Protocol No. 57273316.1.0000.5020). Informed consent was obtained from all parents/guardians before data collection.

## Results

Initially, 528 students aged 12 years were invited to participate in the study. The nonresponse rate was 16% (N = 86). In addition, 13 schoolchildren were not assessed for eligibility because they were not in the school when the informed consent was delivered. Then, the 442 remaining children were assessed for inclusion. Of them, 27 (6.1%) children were excluded due to current use of orthodontic appliances and 15 (3.6%) were excluded from the analysis because of incomplete data. The final sample included 400 participants.

The majority of the sample were female children and from families with monthly family income between ½ and 1 Brazilian minimum wage. Parents/guardians education was predominantly between 8 and 11 years of schooling. The mean of decayed teeth and number of teeth with clinical consequences of untreated dental caries was 0.87 and 0.28, respectively. The prevalence of dental pain in the last 6 months was 36.0%. The mean scores of SOC, social support and CPQ_11-14_ were 45.7, 141.0 and 16.1, respectively (Table 1).

**Table 1:** Sociodemographic characteristics, dental caries measures, symptom status, psychosocial factors and OHRQoL of the studied sample (n = 400)

Variables	N [%(95% CI)]/mean (95% CI)
*Sociodemographic characteristics*	
Sex, N (%)	
Female	231 [57.8 (52.8–62.5)]
Male	169 [(42.2 (7.5–47.2)]
Parents/guardians years of schooling, N (%)	
1–7 years	62 [15.5 (12.3–19.4)]
8–11 years	290 [72.5 (67.9–76.7)]
≥ 12 years	48 [12.0 (9.2–15.6)]
Family income, N (%)	
Up to ½ BMW^A^	103 [25.8 (21.7–30.3)]
½ to 1 BMW	161 [40.2 (35.5–45.2)]
> 1 BMW	136 [34.0 (29.5–38.8)]
*Dental caries measures*	
Decayed teeth, mean (SD)	0.9 (0.7–1.0)
PUFA/pufa score, mean (SD)	0.3 (0.2–0.4)
*Symptom status*	
Dental pain, N (%)	
Yes	144 [36.0 (31.4–40.8)]
No	256 [64.0 (59.2–68.6)]
*Psychosocial factors*	
Sense of coherence, mean (SD)	45.7 (45.0–46.3)
Social support (SSA), mean (SD)	
Total score	141.0 (17.6)
Friends	33.0 (5.7)
Family	42.0 (5.7)
Teachers	29.6 (5.4)
Others	36.5 (5.7)
*Oral health-related quality of life*	
CPQ_11-14_	
Total score, mean (SD)	16.1 (15.3–17.0)
Oral symptoms, mean (SD)	4.5 (4.3–4.8)
Emotional state, mean (SD)	3.4 (3.2–3.7)
Functional limitations, mean (SD)	4.5 (4.3–4.8)
Social well-being, mean (SD)	3.7 (3.4–4.0)

**Fig. 2:**
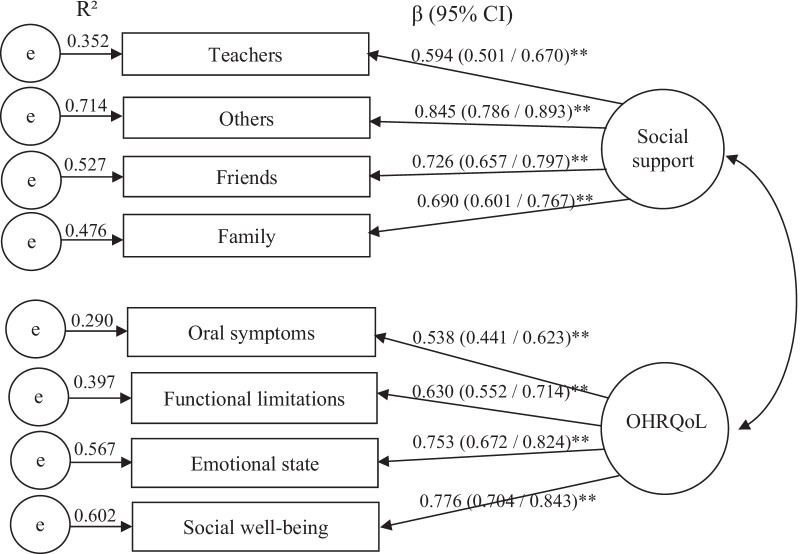
Confirmatory factor analysis of the 2-factor 8 items (measurement model) obtained through bootstrap (standard error/bias-corrected 95% CI). **P < 0.01

The confirmatory factor analysis (CFA) supported the latent variables OHRQoL and social support is presented in Fig. 2. The factor loadings of the items that confirmed the latent variable social support were: teachers (β = 0.594), others (β = 0.845), friends (β = 0.726) and family (β = 0.690). The factor loadings of the items confirming the latent variable OHRQoL were: symptoms (β = 0.538), function (β = 0.630), emotional (β = 0.753) and social (β = 0.776). The highest R^2^ was social support—others (0.714), whereas the lowest R^2^ was CPQ_11-14_—symptoms (0.290).

The fit indices of the measurement model and structural model are presented in Table 2. The CFA and SEM supported the hypothesized measurement model and structural model since both models show good fit, meeting all our priori criteria.

**Table 2:** Fit indices for the measurement and structural models

Model	χ^2^/df	GFI	CFI	SRMR	RMSEA
Measurement model	1.696	0.983	0.988	0.0352	0.042
Structural model	1.935	0.963	0.961	0.0409	0.048

χ^2^ (d.f) (P), chi-square and degrees of freedom; GFI, goodness-of-fit-statistics; CFI, comparative fit index; SRMR, standardised root-mean-squared residual; RMSEA, root-mean-square error of approximation

**Fig. 3:**
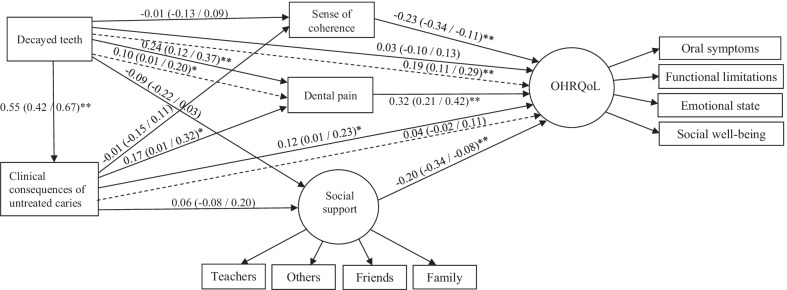
Full model of associations between decayed teeth, clinical consequence of untreated caries, dental pain, SOC, social support and OHRQoL. *P < 0.05; **P < 0.01. The model was adjusted for sex and monthly family income. Direct effects are represented through solid lines, and indirect effects are indicated by dashed lines

The direct and indirect relationships between decayed teeth, clinical consequences of untreated caries, SOC, dental pain, social support and OHRQoL are reported in Fig. 3. Number of decayed teeth directly predicted clinical consequences of untreated caries (β = 0.55) and dental pain (β = 0.24). Clinical consequences of untreated caries were directly linked with dental pain (β = 0.17) and OHRQoL (β = 0.12). OHRQoL was directly predicted by SOC (β = − 0.23), dental pain (β = 0.32) and social support (β = − 0.20). Number of decayed teeth indirectly predicted OHRQoL (β = 0.19) via clinical consequences of untreated caries, SOC, dental pain and social support (Fig. 3).

The different pathways between number of decayed teeth and OHRQoL are described in Table 3. Clinical consequences of untreated caries, dental pain, SOC and social support mediated the relationship between number of decayed teeth and OHRQoL.

**Table 3:** Indirect effects between number of decayed teeth and OHRQoL

Decayed teeth → clinical consequences of untreated caries → OHRQoL = 0.548 × 0.118 = 0.065
Decayed teeth → clinical consequences of untreated caries → dental pain → OHRQoL = 0.548 × 0.166 × 0.320 = 0.029
Decayed teeth → clinical consequences of untreated caries → SOC → OHRQoL = 0.548 × − 0.011 × − 0.232 = 0.001
Decayed teeth → clinical consequences of untreated caries → social support → OHRQoL = 0.548 × 0.056 × − 0.196 = − 0.006
Decayed teeth → dental pain → OHRQoL = 0.244 × 0.320 = 0.078
Decayed teeth → SOC → OHRQoL = − 0.012 × − 0.242 = 0.002
Decayed teeth → Social support → OHRQoL = − 0.088 × − 0.196 = 0.017

The unadjusted and adjusted associations of number of dental caries and clinical consequences of untreated caries with OHRQoL are shown in Table 4. Models 1 showed that dental caries measures were statistically associated with OHRQoL. In models 2, the association between dental caries measures and OHRQoL was adjusted for SOC, social support and dental pain. Dental caries measures remained associated with OHRQoL after adjustment in models 2. The models 3 tested the moderator effect of SOC, dental pain, and social support on the relationship of dental caries and OHRQoL by inserting the interaction term of dental caries and SOC, dental pain and social support. The interaction terms were not associated with OHRQoL, suggesting the absence of moderator effect.

**Table 4:** Multivariable negative binominal regression models on the relationship of number of decayed teeth and clinical consequences of dental caries with OHRQoL

	Model 1 β (95% CI)	Model 2 β (95% CI)	Model 3 β (95% CI)
*Number of decayed teeth and SOC*			
Number of decayed teeth	0.06 (0.03/0.09)**	0.05 (0.02/0.08)**	0.13 (− 0.09/0.35)
SOC	− 0.03 (− 0.04/− 0.02)**	− 0.03 (− 0.03/− 0.02)**	− 0.03 (− 0.03/− 0.02)**
Number of decayed teeth × SOC			− 0.01 (− 0.01/0.00)
∆Dif^†^ = 0.222; df = 4 (χ^2^, *P* = 0.994)			
*Number of decayed teeth and social support*			
Number of decayed teeth	0.061 (0.03/0.09)**	0.05 (0.02/0.09)**	0.03 (− 0.23/0.24)
Social support	− 0.01 (− 0.01/− 0.01)**	− 0.01 (− 0.01/− 0.01)**	− 0.01 (− 0.01/− 0.01)**
Number of decayed teeth × social support			0.00 (− 0.00/0.00)
∆ Dif^†^ = 0.096; df = 4 (χ^2^, *P* = 0.999)			
*Number of decayed teeth and dental pain*			
Number of decayed teeth	0.06 (0.03/0.09)**	0.02 (− 0.01/0.05)	0.04 (0.00/0.09)*
Dental pain	0.14 (0.10/0.18)**	0.13 (0.09/0.17)**	0.16 (0.11/0.21)**
Number of decayed teeth × dental pain			− 0.02 (− 0.04/0.00)
∆ Dif^†^ = 1.425; df = 4 (χ^2^, *P* = 0.840)			
*Clinical consequences of untreated caries and SOC*			
Clinical consequences of untreated caries	0.12 (0.05/0.20)**	0.11 (0.05/0.18)**	0.11 (− 0.28/0.51)
SOC	− 0.03 (− 0.04/− 0.02)**	− 0.03 (− 0.03/− 0.02)**	− 0.03 (− 0.04/− 0.02)**
Clinical consequences of untreated caries × SOC			− 0.00 (− 0.01/0.01)
∆ Dif = 0.001; df = 4 (χ^2^, *P* = 0.999)			
*Clinical consequences of untreated caries and social support*			
Clinical consequences of untreated caries	0.12 (0.05/0.20)**	0.13 (0.06/0.20)**	− 0.18 (− 0.67/0.32)
Social support	− 0.01 (− 0.01/− 0.01)**	− 0.01 (− 0.01/− 0.01)**	− 0.01 (− 0.01/− 0.01)**
Clinical consequences of untreated caries × social support			0.00 (− 0.00/0.01)
∆ Dif = 0.007; df = 4 (χ^2^, *P* = 0.999)			
*Clinical consequences of untreated caries and dental pain*			
Clinical consequences of untreated caries	0.12 (0.05/0.20)**	0.05 (− 0.02/0.12)	0.09 (− 0.00/0.18)
Dental pain	0.14 (0.10/0.18)**	0.13 (0.09/0.17)**	0.14 (0.10/0.18)**
Clinical consequences of untreated caries × dental pain			− 0.02 (− 0.05/0.01)
∆Dif^†^ = 0.811; df = 4 (χ^2^, *P* = 0.937)			

Model 1: unadjusted

Model 2: adjusted for dental caries, sex, monthly family income, SOC, social support and dental pain

Model 3: model 2 + interaction term (dental caries × SOC, dental caries × social support and dental caries × dental pain)

*P < 0.05 and **P ≤ 0.001 are considered significant

^†^Interaction was assessed through comparison likelihood ratio between models 2 and 3

## Discussion

This study examined the possible mediation and moderation effect of SOC, dental pain and social support on the relationship between dental caries and OHRQoL according to explicit hypothesized model based on the Wilson and Cleary theoretical model. The SEM revealed that number of decayed teeth and clinical consequences of untreated caries predicted OHRQoL through indirect or direct effects, respectively. There was a significant indirect effect of number of decayed teeth on OHRQoL that was mediated by SOC, dental pain and social support, suggesting that dental caries impact on OHRQoL through symptoms and psychosocial factors in 12-year-old children. However, severe dental caries assessed using PUFA/pufa index only predicted OHRQoL through direct effects. The possible moderator effect of SOC, dental pain and social support on the relationship of dental caries and clinical consequences of untreated caries with OHRQoL was not detected when both dental caries measures were assessed.

Similar to our findings, psychosocial factors, including SOC and social support have been linked with OHRQoL [3–6]. A recent systematic review also highlighted that those psychosocial factors were relevant protective predictors of dental caries in children and adolescents [22]. Of them, high social support and greater SOC were inversely associated with dental caries in those age groups [22]. Therefore, our findings on the mediation effect of SOC and social support on the relationship between dental caries and OHRQoL support the salutogenenic theory that explain how individual psychological characteristics facilitate the ability to cope effectively with the difficulties during daily life, favouring better oral clinical conditions and consequently better OHRQoL.

The adequacy of the structural equation models supported the application of Wilson and Cleary theoretical framework to investigate the predictors and mediators of OHRQoL involving 12-year-old children. Previous studies also revealed that Wilson and Cleary is a suitable model in research involving quality of life amongst children [23, 24], adolescents [4, 6, 25] and adults [26, 27]. The present study also benefits from the robust statistical methods, including SEM to simultaneously analyze the direct and indirect relationships between variables and the use of negative binomial regression to assess moderator effects.

Few studies concurrently assessed the impact of both untreated dental caries and their sequelae that remain untreated on OHRQoL in schoolchildren aged 2–4 years [28], 2–5 years [29], 8–10 years old [30], and 12 years old [15]. According to our findings and previous research, untreated dental caries and their clinical consequence exerted a negative impact on children’s OHRQoL [28–30]. However, as far as the authors are aware, no previous studies concomitantly assessed the role of mediators and moderators of effect between untreated dental caries and their sequelae that remain untreated with OHRQoL.

Dental pain has been reported as a mediator of the impact of dental caries on OHRQoL [4, 31, 32]. Evidence on the importance of psychosocial factors as mediators in the relationship between dental caries and OHRQoL is scarce notwithstanding. Overall, most studies evaluated the direct and indirect effect of psychosocial factors on OHRQoL [3, 6, 23, 27].

Dental pain, SOC, and social support were mediators on the link between number of teeth with dental caries and OHRQoL. This finding has implications for clinical practice and health promotion actions. For instance, improving symptoms status and psychosocial factors along with dental restoration may improve children’s clinical status and OHRQoL into a greater extent than providing dental restorations only. Possibly, a multi-professional approach involving oral health professionals and psychologists would potentially benefit children’s OHRQoL. Previous studies showed positive effect of intervention to enhance SOC or social support to improve OHRQoL [33, 34]. So, investigations whether dental treatment combined with psychosocial interventions would result in better OHRQoL than offering only one type of intervention is a potential topic for future intervention studies. The lack of moderator effect of SOC, dental pain and social support on the relationship between dental caries and OHRQoL indicate that dental caries impact on children’s OHRQoL regardless of the child’s level of SOC, dental pain and social support. Thus, our findings support that population approaches should be used to improve children’s oral health since this age group would benefit from those strategies whatsoever are their symptom status and psychosocial levels.

Some limitations of this study should be considered. First, the cross-sectional design restricts the interpretation of causal processes underlying the associations hypothesized in the theoretical model. Second, the studied sample has specific characteristics, including their age and the low socioeconomic level. These aspects prevent extrapolating our findings to other age groups and those from different socioeconomic status.

In addition, the levels of dental caries in the sample can be considered relatively low when compared with children in the same age examined in the last oral health survey in Brazil in 2010 [35]. For instance, the mean of the number of decayed teeth was 0.87 in the studied sample, and 1.12 among 12-year-old children in country and 1.49 in the city of Manaus according to the national oral health survey in 2010. Also, the prevalence of dental pain during the last 6 months was 36% in our sample, which was higher than the prevalence observed among 12-year-old children in the last oral health survey (25%) [35]. The global decline in dental caries over the last years in both developed and developing countries and the increase of oral health disparities may explain this finding [36].

One of the great challenges of oral health is to effectively respond to the most prevalent oral problems in the population. In this study, the indirect relationships between dental caries and OHRQoL mediated by symptoms and psychosocial factors indicate that strategies aiming at improving children’s quality of life should consider dental treatment through clinical procedures accompanied by educational activities and health promotion strategies of expanded, updated and differentiated scope. Thus, the present findings make an important contribution to clinical decision making and priority setting in public oral health care.

## Conclusion

Our results suggest that clinical consequences of untreated caries was directly associated with OHRQoL amongst 12-yeard-old schoolchildren from a region with low socioeconomic indicators in Brazil. Moreover, symptom status (dental pain) and psychosocial factors were important mediators on the link between dental caries and OHRQoL in schoolchildren. The moderator effect of symptom status (dental pain) and psychosocial factors on the abovementioned relationship was not observed. Future studies should investigate whether oral health care combining dental treatment and psychological interventions would reduce the impact of dental diseases on children’s OHRQoL.

## Data Availability

The data that support the findings of this study are available from the Dental School, Federal University of Amazonas but restrictions apply to the availability of these data, which were used under license for the current study, and so are not publicly available. Data are however available from the author Profa Maria Augusta Bessa Rebelo (email: augusta@ufam.edu.br) upon reasonable request and with permission of the Dental School, Federal University of Amazonas.
