# Correction: The role of dental pain and psychosocial factors on the relationship between dental caries and oral health-related quality of life in children

**DOI:** 10.1186/s12903-022-02541-3

**Published:** 2022-11-04

**Authors:** Cilio Antonio Ribeiro Junior, Mario Vianna Vettore, Janete Maria Rebelo Vieira, Ana Paula Corrêa de Queiroz Herkrath, Adriana Corrêa de Queiroz, Juliana Vianna Pereira, Fernando José Herkrath, Maria Augusta Bessa Rebelo

**Affiliations:** 1 School of Dentistry, Federal University of Amazonas, Av. Ministro Waldemar Pedrosa, 1539, Praça 14 de Janeiro, Manaus, AM CEP 69025-050 Brazil; 2 Department of Health and Nursing Sciences, Faculty of Health and Sports Sciences, University of Agder, Campus Kristiansand, Universitetsveien 25, 4630 Kristiansand, Norway; 3 Instituto Leônidas e Maria Deane, Fundacão Oswaldo Cruz, Rua Teresina, 476, Adrianópolis, Manaus, AM CEP: 69027-070 Brazil

**Correction: BMC Oral Health, 22 340 (2022)**

**https://doi.org/10.1186/s12903-022-02372-2**

In this article [1] the author’s name Adriana Corrêa de Queiroz was incorrectly written as Adriana Correa de Queiroz Herkrath. This has been corrected with this correction.

The original article has been corrected.
